# Population pharmacokinetic modeling of PF-06439535 (a bevacizumab biosimilar) and reference bevacizumab (Avastin^®^) in patients with advanced non-squamous non-small cell lung cancer

**DOI:** 10.1007/s00280-019-03946-8

**Published:** 2019-11-26

**Authors:** Cheryl S. W. Li, Kevin Sweeney, Carol Cronenberger

**Affiliations:** 1 Clinical Pharmacology/Pharmacometrics, Pfizer Global Product Development, Pfizer Inc., 300 Technology Square, Cambridge, MA 02140 USA; 2 Clinical Pharmacology/Pharmacometrics, Pfizer Global Product Development, Pfizer Inc., Eastern Point Road, Groton, CT 06340 USA; 3 Clinical Pharmacology/Pharmacometrics, Pfizer Global Product Development, Pfizer Inc., 500 Arcola Road, Collegeville, PA 19426 USA

**Keywords:** Bevacizumab, Biosimilar, Non-small cell lung cancer, PF-06439535, Population pharmacokinetics

## Abstract

**Purpose:**

The objectives of this analysis were to characterize the population pharmacokinetics (PK) of PF-06439535 (a bevacizumab biosimilar) and reference bevacizumab (Avastin^®^) sourced from the European Union (bevacizumab-EU) in patients with advanced non-squamous non-small cell lung cancer (NSCLC), and to quantify the difference in PK parameters between the two drug products via covariate analysis.

**Methods:**

Pooled PF-06439535 and bevacizumab-EU serum concentration data from a comparative clinical efficacy and safety study (NCT02364999) in patients with NSCLC (*N* = 719) were analyzed using a non-linear mixed-effects modeling approach. Patients received PF-06439535 plus chemotherapy or bevacizumab-EU plus chemotherapy every 21 days for 4–6 cycles, followed by monotherapy with PF-06439535 or bevacizumab-EU. PF-06439535 or bevacizumab-EU was administered intravenously at a dose of 15 mg/kg. Effects of patient and disease covariates, as well as the drug product (PF-06439535 versus bevacizumab-EU), on PK were investigated.

**Results:**

Overall, 8632 serum bevacizumab concentrations from 351 patients in the PF-06439535 group and 354 patients in the bevacizumab-EU group were included in the analysis. A two-compartment model adequately described the combined data. Clearance (CL) and central volume of distribution (*V*_1_) estimates were 0.0113 L/h and 2.99 L for a typical 71-kg female patient with NSCLC administered bevacizumab-EU. CL and *V*_1_ increased with body weight and were higher in males than females even after accounting for differences in body weight. The 95% confidence intervals for the effect of drug product on CL and *V*_1_ encompassed unity.

**Conclusions:**

The population PK of PF-06439535 and bevacizumab-EU were well characterized by a two-compartment model. Covariate analysis did not reveal any appreciable differences between PK parameters for PF-06439535 and bevacizumab-EU in patients with NSCLC.

**Clinical trial registration:**

ClinicalTrials.gov, NCT02364999.

**Electronic supplementary material:**

The online version of this article (10.1007/s00280-019-03946-8) contains supplementary material, which is available to authorized users.

## Introduction

Bevacizumab (Avastin^®^) is a recombinant humanized immunoglobulin G1 monoclonal antibody that inhibits angiogenesis by binding to and neutralizing the biological activity of human vascular endothelial growth factor [1, 2]. Although its specific licensed indications vary across countries [1, 2], bevacizumab is used in the treatment of several cancers. In the United States (US), for example, bevacizumab is licensed for the treatment of metastatic colorectal cancer; unresectable, locally advanced, recurrent, or metastatic non-squamous non-small cell lung cancer (NSCLC); recurrent glioblastoma; metastatic renal cell carcinoma; persistent, recurrent, or metastatic cervical cancer; and epithelial ovarian, fallopian tube, or primary peritoneal cancer [2].

Biosimilars are biological products that are highly similar to a licensed reference product in terms of structure, biological activity, pharmacokinetics (PK) and pharmacodynamics (PD), and efficacy, safety, and immunogenicity profile [3–5]. In the US, for example, biosimilarity is defined as meaning that “the [biosimilar] product is highly similar to the reference product notwithstanding minor differences in clinically inactive components”, and that “there are no clinically meaningful differences between the [biosimilar] and the reference product in terms of the safety, purity, and potency of the product” [4].

The bevacizumab biosimilar PF-06439535 (Zirabev™) has an identical primary structure and similar biologic function as compared with reference bevacizumab (Avastin^®^) sourced from the European Union (bevacizumab-EU) and US (bevacizumab-US) [6]. Similarity between PF-06439535 and bevacizumab-EU was also demonstrated in a non-clinical toxicity study in cynomolgus monkeys [6]. In the clinical program, the PK similarity of PF-06439535 to both bevacizumab-EU and bevacizumab-US, and of bevacizumab-EU to bevacizumab-US, was demonstrated in a randomized, three-arm study in healthy males (Study B7391001; NCT02031991) [7]. The PK of the three products was compared after a single intravenous (IV) dose of 5 mg/kg. For the comparisons of PF-06439535 to each of the two bevacizumab reference products, and for the comparison of bevacizumab-EU to bevacizumab-US, the 90% confidence intervals (CIs) for the test-to-reference ratios of maximum observed concentration (*C*_max_), area under the concentration–time curve from time zero to the last quantifiable concentration (AUC_*t*_), and area under the concentration–time curve from time zero to infinity (AUC_inf_) were all within the predefined bioequivalence window of 80.00–125.00% [7].

In addition to the PK similarity study in healthy volunteers, the serum concentrations of PF-06439535 and bevacizumab-EU have been characterized in a comparative clinical study in patients [8]. Study B7391003 was conducted to evaluate the efficacy, safety, PK, and immunogenicity of PF-06439535 versus bevacizumab-EU, when each product was given in combination with paclitaxel and carboplatin in the first-line treatment of advanced non-squamous NSCLC. The primary objective of the study was to assess the similarity of PF-06439535 and bevacizumab-EU by comparing the confirmed objective response rate by Week 19 in each treatment group. Efficacy, safety, immunogenicity, and drug concentration results have been reported previously [8].

The objectives of the current work were to characterize the population PK of PF-06439535 and bevacizumab-EU in Study B7391003, and to evaluate the influence of selected covariates, including the drug product (i.e., PF-06439535 versus bevacizumab-EU), on the PK parameters. To our knowledge, this is the first reported use of a population modeling approach to quantify the potential differences in PK between a bevacizumab biosimilar and reference bevacizumab based on a comparative clinical efficacy and safety study.

## Materials and methods

### Clinical study and PK assessments

Study B7391003 was a multinational, double-blind, randomized, parallel-group clinical trial [8]. The study was registered at ClinicalTrials.gov (NCT02364999) and EudraCT (2014-003878-16). Details regarding the ethical conduct of the study and informed consent can be found in the “Compliance with ethical standards” section.

Between May 2015 and November 2016, patients (*N* = 719) with advanced non-squamous NSCLC were randomized (1:1) to receive first-line treatment with at least four, but not more than six, cycles of either PF-06439535 plus paclitaxel and carboplatin or bevacizumab-EU plus paclitaxel and carboplatin, followed by monotherapy with PF-06439535 or bevacizumab-EU as previously assigned. Randomization was stratified by region (according to the location of the drug depot supplying the site), sex (male/female), and smoking history (never/ever). Monotherapy with PF-06439535 or bevacizumab-EU could continue until disease progression, unacceptable toxicity, or the withdrawal of consent, among other reasons. The expected duration of study participation for individual patients was approximately 1 year.

Blinded PF-06439535 or bevacizumab-EU was administered once at the start of every 21-day cycle. The initial dose was 15 mg/kg delivered over 90 min as an IV infusion. If the first infusion was well tolerated, the second infusion could be delivered over 60 min. If the 60-min infusion was well tolerated, all subsequent infusions could be administered over 30 min.

Serum samples for the determination of bevacizumab (PF-06439535 or bevacizumab-EU) drug concentrations were collected prior to infusions of bevacizumab. In addition, samples were collected 1 h (± 0.5 h) after the end of the infusion on Cycle 1 Day 1 and Cycle 5 Day 1 (if a patient received Cycle 5); samples were also collected during the end-of-treatment visit that occurred after patients had discontinued all study treatment. Serum samples identified in the analysis plan were analyzed for concentrations of PF-06439535 and bevacizumab-EU using a validated, sensitive, and specific enzyme-linked immunosorbent assay. The lower limit of quantification (LLOQ) of the PK assay was 250 ng/mL.

The study was considered to be complete when the last available patient completed up to 1 year from randomization plus 28-day follow-up. The last patient visit was in December 2017.

### Population PK analysis dataset

Overall, 705 patients (351 in the PF-06439535 group and 354 in the bevacizumab-EU group) were randomized and received PF-06439535 or bevacizumab-EU as planned, had no major protocol deviations, and had at least one drug concentration measurement post-drug administration. These patients comprised the PK population for Study B7391003, and data from all 705 patients were included in the original PK dataset. When building the dataset for the population PK analysis, the original PK data were further evaluated with respect to structural data completeness and logical integrity, and 73 PK observations were excluded: 36 were Cycle 1 Day 1 pre-dose samples for patients with significant or discordant pre-dose concentrations (pre-dose concentration > 2 times the LLOQ of 250 ng/mL), and 37 were post-dose serum concentration values that were below the LLOQ. Since the number of post-dose values below the limit of quantification (BLQ) was small (37 BLQ values out of 8004 total post-dose observations; 0.46%), the M1 method [9] was applied, whereby post-dose BLQ observations were excluded from the dataset.

The final data used for the population PK analysis consisted of a total of 8632 serum bevacizumab concentrations, including 4276 observations from 351 patients treated with PF-06439535 and 4356 observations from 354 patients treated with bevacizumab-EU.

### Model-building strategy and software

A population PK model was developed using pooled data from the two treatment groups in Study B7391003. The analysis was conducted using a non-linear mixed-effects modeling approach as follows: base model development, covariate model development, final model development, model qualification (goodness of fit), and validation of the final model.

Population analysis was conducted using NONMEM^®^ software version 7.2 (ICON, Ellicott City, MD, USA). Graphical and other evaluations of NONMEM outputs were performed using R-3.2.2 (R Foundation for Statistical Computing, Vienna, Austria) or RStudio^®^ (RStudio Inc, Boston, MA, USA). Perl-speaks-NONMEM (PsN) version 4.2.0 [10] was used for stepwise covariate modeling (SCM), visual predictive check (VPC), and non-parametric bootstrapping. The NONMEM first-order conditional estimation method with *η*–*ε* interaction (where *η* is the empirical Bayes prediction of the inter-individual random effect in a PK parameter and *ε* is the residual variability in NONMEM) was employed for all model runs.

### Base model and random-effects model development

Based on reported population PK analyses of reference bevacizumab [11, 12] and the observed bi-exponential serum concentration–time profiles of PF-06439535 and reference bevacizumab in Study B7391001 [7], a two-compartment structural PK model with zero-order input (constant-rate IV infusion) and first-order elimination from the central compartment was used as the starting structural model. Since body weight was a significant covariate impacting both clearance (CL) and central volume of distribution (*V*_1_) in previous analyses of reference bevacizumab [11, 12], and administration of PF-06439535 or bevacizumab-EU was normalized for body weight (i.e., 15 mg/kg in Study B7391003), the effect of body weight on CL and *V*_1_ was incorporated into the structural model to improve model stability.

Assuming a log-normal distribution, the inter-individual variance (IIV) in PK parameters, including CL, intercompartmental clearance (*Q*), *V*_1_, and peripheral volume of distribution (*V*_2_) was described by an exponential model, as presented in Eq. (1):

1 $${P_j} = {\rm{TVP}} \times e_{}^{{\eta _j}},$$

where *P*_*j*_ is the individual value of the PK parameter in the *j*th patient, TVP is the typical value of the parameter in the population, and *η*_*j*_ is a random effect with a mean of zero and variance of *ω*^2^. The IIV was only estimated for the central compartment PK parameters (i.e., CL and *V*_1_) because the relative standard error for *Q* was > 100% when the IIV was estimated either for all four central and peripheral compartment PK parameters (CL, *Q*, *V*_1_, and *V*_2_) or for CL, *V*_1_, and *V*_2_. This suggested that the sparse PK data from the study did not support the estimation of IIV on the peripheral compartment PK parameters; i.e., *V*_2_ and *Q*.

The vector of inter-individual random effects (across parameters) has the variance–covariance matrix *Ω*. Since no significant correlations between CL and *V*_1_ were observed with a diagonal *Ω* matrix during model development, the diagonal structure was implemented to obtain a stable model given the sparse nature of the data.

The residual error was described using an additive error model after log-transforming the PK data. The residual variability in PF-06439535 and bevacizumab-EU concentrations was modeled using the following model structure:

2 $$ \ln (Y_{ij} ) = \ln (F_{ij} ) + W \times \varepsilon_{kij} , $$

where *Y*_*ij*_ is the observed PF-06439535 and bevacizumab-EU serum concentration value in the *j*th patient at the *i*th time point, *F*_*ij*_ is the corresponding model-predicted value, and *ε*_*kij*_ is the corresponding residual error for the *k*th term, normally distributed with mean zero and variance $\sigma_{i}^{2}$ of 1. *W* is the estimated residual variance. Diagnostic plots were reviewed to ensure the adequacy of the fit. The result of this stage of model development was considered the final base model.

### Covariate model development

Following base model development, inclusion of covariates was evaluated using the SCM method. The covariates explored for CL and *V*_1_ included those reported in the regulatory labels [1, 2] and in the literature [11, 12] for reference bevacizumab. The influence of the following covariates was assessed: Asian versus non-Asian race (including Japanese versus non-Japanese designation), baseline body weight, sex, baseline albumin, baseline alanine aminotransferase, baseline alkaline phosphatase, baseline anti-drug antibody (ADA) status, baseline Eastern Cooperative Oncology Group performance status, number of metastatic sites, longest tumor diameter, and drug product (PF-06439535 versus bevacizumab-EU). Evaluation of the covariates to be included in the SCM analysis was based on visual inspection of relationships between *η* on CL and *V*_1_ and the covariates, the robustness of the covariate data, and the specific objectives of the PK analysis.

Continuous covariates were modeled using a power model as described in the following equation:

3 $$ {\text{TVP}}_{j} = P_{\text{pop}} \times \left( {\frac{{{\text{COV}}_{j} }}{{{\text{COV}}_{\text{median}} }}} \right)^{\theta } , $$

where TVP_*j*_ represents the model-predicted PK parameter for the typical *j*th individual with normalized covariate value (COV_*j*_/COV_median_), *P*_pop_ represents the population central tendency for the PK parameter at the median covariate value, and *θ* represents the estimated scale factor.

Most categorical covariates (e.g., Japanese versus non-Japanese or number of metastatic sites) were modeled using the general equation:

4 $$ {\text{TVP}}_{j} = P_{\text{pop}} \times (1 + \theta ), $$

where, for the X groups within a given category,

if COV = group 1 (most common), *θ* = 0,

if COV = group 2, *θ* = *θ*_1_,

…

if COV = group X, *θ* = *θ*_*x*−1_.

The drug product effect (PF-06439535 versus bevacizumab-EU) parameterized as a categorical covariate was modeled using the general equation below for improved model stability:

5 $$ {\text{TVP}}_{j} = \theta^{\text{COV}} , $$

where, for the groups within a given category,

if COV = group 1, COV = 0, TVP_*j*_ = 1

if COV = group 2, COV = 1, TVP_*j*_ = *θ*.

Overall, the categorical covariate effects were modeled as a fractional change (Eqs. 4 and 5).

Inclusion of covariates in the PK model was based on the likelihood ratio test to compare nested models and was implemented in a forward inclusion/backward elimination SCM procedure. A pre-specified value of *α* = 0.05 was implemented during the forward selection process to assess the significance of including a covariate in the model in a stepwise fashion. The test for elimination of a covariate, given that others were kept in the model, was performed at a pre-specified significance level of *α* = 0.001. Furthermore, to obtain the most parsimonious and stable final model, the candidate covariate model resulting from the backward elimination step in SCM was subjected to a separate NONMEM run with $COV step executed to examine any sign of model overparameterization and poorly estimated parameters.

### Outliers

During final model development, data were classified as outliers using the conditional population weighted residuals (CWRES) and individual weighted residuals (IWRES). Data with |CWRES| > 6 or |IWRES| > 6 were considered potential outliers. The influence of these potential outliers was evaluated by comparing estimates of the key model parameters (e.g., CL, *V*_1_) from model fits of data with and without the outliers. The outliers were considered influential if the parameter estimates differed by more than 20%, and in such cases subsequent model development was performed without the outlying observations.

### Assessment of model adequacy (goodness of fit)

Goodness of fit of different models to the data was evaluated using the following criteria: change in objective function value (OFV), condition number, visual inspection of different diagnostic plots, precision of the parameter estimates, and decreases in IIV and residual variance. At all stages of model development, diagnostic plots were examined to assess model adequacy, possible lack of fit, or violation of assumptions. Plots of observed values versus population predicted values, and observed values versus individual predicted values, were evaluated for randomness around the line of unity. The longitudinal profiles of PK concentrations were evaluated for comparison of observations and predictions. Plots of CWRES versus time and CWRES versus concentration were evaluated for randomness around the zero line. Distribution of *η* was checked to ensure approximately normal distribution. In addition, plots of *η* versus each covariate were evaluated for the base model and the final model to demonstrate that the final model accounted for trends observed with the base model. A CI was constructed for each parameter based on non-parametric bootstrapping (1000 bootstrap datasets).

### Assessment of model predictive performance (validation)

An assessment as to whether the final model described the central tendency and variability in the observed data was evaluated by a VPC. The VPC was conducted by simulating concentrations for 1000 trials of the same trial design using the original datasets (e.g., dosing records, observation times, covariate values) and the final PK model, and calculating and comparing the median and quantiles of the observed data to the quantiles of the simulated data. The concordance between the central tendency and variability of the observed and simulated concentrations was evaluated. The 2.5th and 97.5th percentiles and the median for the observed data were calculated and presented with the corresponding percentiles for the simulated data.

## Results

### Observed PK

Baseline characteristics of patients in the two treatment groups are summarized in Table 1. The PK population was predominantly non-Asian (89% of patients), with a median weight of 71 kg. The distribution of covariates was similar between the two treatment groups.

**Table 1:** Summary of baseline characteristics by treatment group (PK population)

	All (*N* = 705)	PF-06439535 (*N* = 351)	Bevacizumab-EU (*N* = 354)
Continuous covariates, median (range)			
Body weight (kg)	71.0 (28.0–135)	70.0 (40.0–132)	72.0 (28.0–135)
Alanine aminotransferase (U/L)	19.0 (3.00–119)	19.3 (3.98–119)	18.0 (3.00–108)
Albumin^a^ (mg/dL)	4.05 (2.10–5.80)	4.00 (2.10–5.70)	4.10 (2.40–5.80)
Alkaline phosphatase^b^ (U/L)	114 (30.0–999)	113 (43.0–954)	114 (30.0–999)
Longest tumor diameter^c^ (mm)	60.0 (10.0–297)	59.0 (10.0–279)	61.0 (10.0–297)
Categorical covariates, *n* (%)			
Drug product			
PF-06439535	351 (49.8)	351 (100)	0 (0.00)
Bevacizumab-EU	354 (50.2)	0 (0.00)	354 (100)
Sex			
Male	457 (64.8)	232 (66.1)	225 (63.6)
Female	248 (35.2)	119 (33.9)	129 (36.4)
Race			
White	625 (88.7)	312 (88.9)	313 (88.4)
Black	4 (0.567)	3 (0.855)	1 (0.282)
Asian	75 (10.6)	36 (10.3)	39 (11.0)
Other	1 (0.142)	0 (0.00)	1 (0.282)
Japanese			
Yes	19 (2.70)	8 (2.28)	11 (3.11)
No	686 (97.3)	343 (97.7)	343 (96.9)
ADA status (prior to treatment)^d^			
Positive	4 (0.575)	1 (0.289)	3 (0.860)
Negative	692 (99.4)	346 (99.7)	346 (99.1)
Number of metastatic sites			
0	52 (7.38)	21 (5.98)	31 (8.76)
1	256 (36.3)	131 (37.3)	125 (35.3)
2	234 (33.2)	118 (33.6)	116 (32.8)
3	111 (15.7)	56 (16.0)	55 (15.5)
4	38 (5.39)	17 (4.84)	21 (5.93)
5	11 (1.56)	6 (1.71)	5 (1.41)
6	3 (0.426)	2 (0.570)	1 (0.282)
ECOG status			
0	203 (28.8)	94 (26.8)	109 (30.8)
1	502 (71.2)	257 (73.2)	245 (69.2)

*ADA* anti-drug antibody, *bevacizumab*-*EU* reference bevacizumab sourced from the European Union, *ECOG* Eastern Cooperative Oncology Group, *PK* pharmacokinetics

^a^Baseline albumin data missing for one patient (bevacizumab-EU group)

^b^Baseline alkaline phosphatase data missing for one patient (bevacizumab-EU group)

^c^Baseline longest tumor diameter data missing for 29 patients (PF-06439535 group) and 18 patients (bevacizumab-EU group)

^d^Baseline ADA not evaluated for four patients (PF-06439535 group) and five patients (bevacizumab-EU group)

**Fig. 1:**
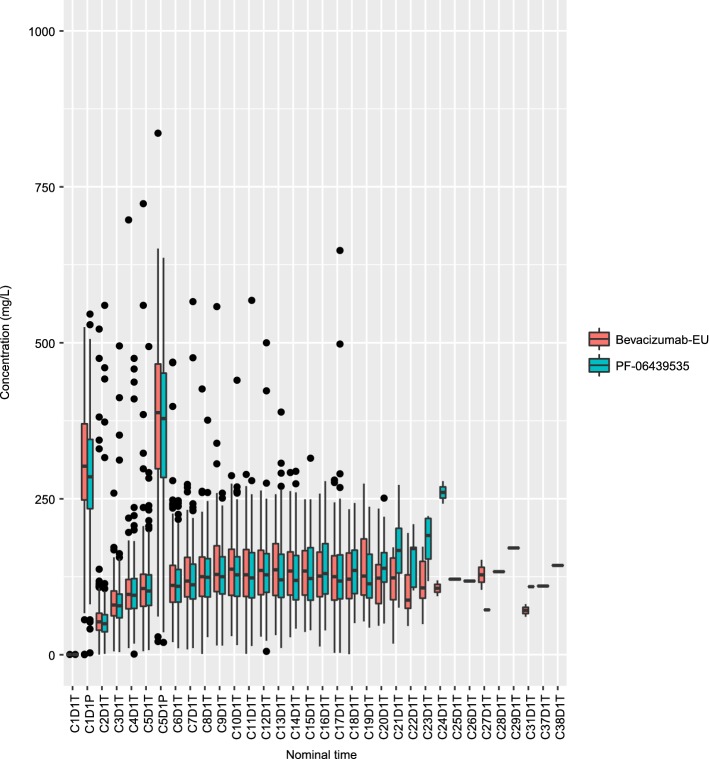
Box plots of observed serum bevacizumab concentrations by treatment group (PF-06439535 or bevacizumab-EU) in the final NONMEM analysis dataset. Individual box plots represent the median (horizontal line) and 25%/75% quartiles, with whiskers extending to the last data point within 1.5 times the interquartile range. Outliers are indicated by solid circles. *Bevacizumab*-*EU* reference bevacizumab sourced from the European Union, *C* cycle, *D* day, *NONMEM* non-linear mixed-effects modeling, *P* peak concentration, *T* trough concentration

The observed serum bevacizumab concentrations were plotted against nominal sampling time by treatment group, as shown in Fig. 1. The concentration–time profiles appeared to be similar between the PF-06439535 and bevacizumab-EU treatment groups.

### Base model development and covariate assessment

A two-compartment model with zero-order input (constant-rate infusion) and first-order elimination from the central compartment, incorporating the effects of baseline body weight on CL and *V*_1_, adequately described the data. A decrease of 1089 in OFV was observed when fitting the two-compartment model versus a one-compartment model.

Population PK parameter estimates for the base model are provided in Online Resource, Table S1. The base model achieved satisfactory precision for parameter estimates, and the diagnostic plots indicated overall reasonable fit of the model to the data (data not shown).

**Fig. 2:**
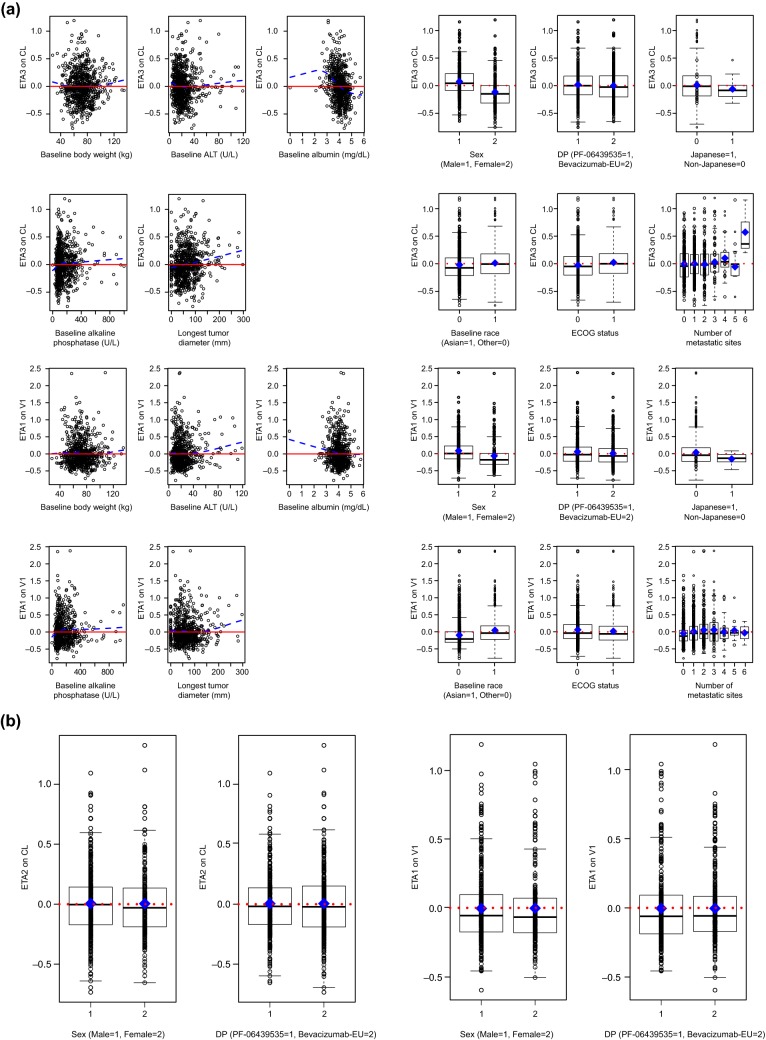
Relationships between baseline covariates of interest and *η* (ETAs) on CL and *V*_1_ in **a** the base model and **b** the final model for PF-06439535 and bevacizumab-EU. In the plots of ETAs versus continuous covariates, each dashed blue line represents a locally estimated scatter plot smoothing line. In the plots of ETAs versus categorical covariates, individual box plots represent the median (horizontal line), mean (blue diamond), and 25%/75% quartiles, with whiskers extending to the last data point within 1.5 times the interquartile range. *ALT* alanine aminotransferase, *bevacizumab*-*EU* reference bevacizumab sourced from the European Union, *CL* systemic clearance, *DP* drug product, *ECOG* Eastern Cooperative Oncology Group, *η/ETA* empirical Bayes prediction of the inter-individual random effect in a pharmacokinetics parameter, *V*_*1*_ volume of distribution in the central compartment

Figure 2a shows the relationships between baseline covariates of interest and *η* on CL and *V*_1_ of the base model for PF-06439535 and bevacizumab-EU. After correcting for baseline body weight in the structural model, CL and *V*_1_ appeared to be slightly higher in males. Some minor visual correlations were observed between the PK parameters and baseline albumin, alanine aminotransferase, alkaline phosphatase, and longest tumor diameter.

Based on visual examination of the correlations, covariates that were tested using the SCM included the continuous variables of baseline albumin, alanine aminotransferase, alkaline phosphatase, and longest tumor diameter, and the categorical variable of sex. Additionally, although no visual relationship between drug product (PF-06439535 versus bevacizumab-EU) and *η* was observed, the categorical variable of drug product was included in the SCM and retained in the final model because the main objective of this work was to understand whether the population PK of PF-06439535 is consistent with that of bevacizumab-EU in patients with NSCLC. By including this variable in the final model, a quantitative assessment of the impact of drug product on PK parameters was made.

Baseline ADA status was not tested as a covariate given the very low incidence observed in the study. In addition, Asian versus non-Asian race and Japanese versus non-Japanese designation were not tested as covariates because the patients in Study B7391003 were predominantly non-Asian (89%) and non-Japanese (97%). In a previous analysis, reference bevacizumab PK was reported to be similar between Asian and non-Asian patients [11], suggesting that the effects of these demographic groups on PK, if any, are likely to be small; the data from the current study were not robust enough for such evaluation.

A summary of the covariate evaluation is provided in Online Resource, Table S2. Following the SCM analysis (after forward selection, *α* = 0.05), the effects of sex on CL and *V*_1_ were included in the full model. Following the backward elimination procedure (*α* = 0.001), the effects of sex on CL and *V*_1_ were retained in the final model.

### Final PK model

When the final model analysis was initially conducted using all data, 27 concentrations from 23 patients had |CWRES| > 6 or |IWRES| > 6. These concentrations accounted for a small number of post-dose data points (27 of 7967, ~ 0.34%) and, in most cases, represented a single inconsistent concentration in a patient (e.g., trough concentration at a visit substantially different from the trough concentration at adjacent visits). A sensitivity analysis was performed using a separate dataset excluding these 27 concentrations and it was determined that they were influential on the PK parameter estimates (i.e., exclusion led to > 20% change from original estimates of the PK parameters, particularly *V*_1_). Therefore, in accordance with the pre-specified population PK analysis plan for Study B7391003, these 27 influential concentrations were omitted from the analysis, and the final population PK model was refitted to the data.

**Fig. 3:**
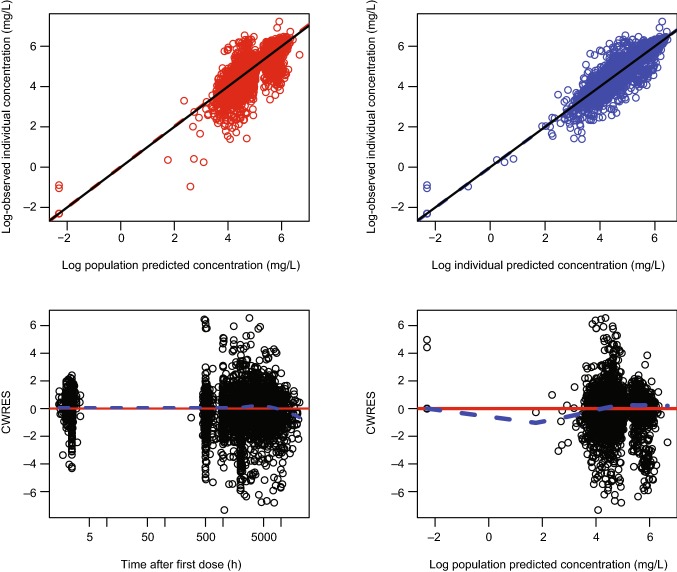
Goodness of fit plots for the final PF-06439535 and bevacizumab-EU model. In the scatter plots of observations versus predictions, the solid line shows the reference line (diagonal line) and the dashed line shows the linear regression line based on the individual data points. In the scatter plots of residuals, the solid line and dashed line show the reference line (*y* = 0) and locally weighted scatter plot smoothing trend line, respectively. Observed concentrations and individual predictions were log-transformed. *Bevacizumab*-*EU* reference bevacizumab sourced from the European Union, *CWRES* conditional population weighted residuals

Final model parameters were estimated with acceptable precision (Table 2). Figure 3 depicts prediction- and residual-based diagnostic plots for the final model. No systematic bias or lack of fit was observed.

**Table 2:** Parameter estimates and confidence intervals from the final model and bootstrap analysis for PF-06439535 and bevacizumab-EU

Parameter	NONMEM results OFV = − 3488.135			Non-parametric bootstrap		
	Estimate	SE	Shrinkage (%)	Estimate (median)	95% CI^a^	
					Lower	Upper
*V*_1_ (L)	2.99	0.373	–	2.96	1.92	3.43
*V*_2_ (L)	6.09	0.812	–	6.15	4.61	7.45
CL (L/h)	0.0113	0.000384	–	0.0113	0.0105	0.0120
*Q* (L/h)	0.269	0.272	–	0.262	0.0411	0.767
BWT effect on *V*_1_	0.468	0.152	–	0.504	0.315	0.815
BWT effect on CL	0.354	0.0591	–	0.352	0.241	0.474
Sex effect on *V*_1_	0.247	0.0411	–	0.264	0.152	0.480
Sex effect on CL	0.262	0.0342	–	0.262	0.199	0.332
Drug product effect on ${ \rm{V}_1^{\rm{b}}}$	1.07	0.0369	–	1.07	0.995	1.18
Drug product effect on CL^b^	1.02	0.0245	–	1.02	0.973	1.07
$\omega_{{V_{1} }}^{2}$	0.117	0.0478	23.4	0.117	0.0745	0.317
$\omega_{\text{CL}}^{2}$	0.0871	0.00726	6.74	0.0857	0.0724	0.101
Residual additive error	0.284	0.00733	6.15	0.284	0.269	0.298

*Bevacizumab*-*EU* reference bevacizumab sourced from the European Union, *BWT* body weight, *CI* confidence interval, *CL* systemic clearance, *NONMEM* non-linear mixed-effects modeling, *OFV* objective function value, *Q* intercompartmental clearance, *SE* standard error, *V*_*1*_ volume of distribution in the central compartment, *V*_*2*_ volume of distribution in the peripheral compartment

Parameters that were not applicable are represented with dashes

^a^The bootstrap runs that had successful minimization (1000 out of 1000) were included in the calculation of the 95% CI. The 95% CI represents 2.5–97.5th percentiles of the included bootstrap estimates

^b^The drug product variable was retained in the final model for the 95% CI estimate despite no statistical effects on CL or *V*_1_ based on the stepwise covariate modeling analysis

The equations below describe the final population parameter estimates for CL and *V*_1_:

$${\rm{CL}} = 0.0113 \, {\mkern 1mu} {\rm{L}}/{\rm{h}} \times {\left( {{{{\rm{BWT}}} \over {71}}} \right)^{0.354}} \times \left( {1.262{\mkern 1mu}\, {\rm{in}}{\mkern 1mu}\, {\rm{male}}} \right) \times \left( {1.02\,{\mkern 1mu} {\rm{in}}\,{\mkern 1mu} {\rm{PF-}}06439535} \right),$$

$${V_1} = 2.99{\mkern 1mu} \,{\rm{L}} \times {\left( {{{{\rm{BWT}}} \over {71}}} \right)^{0.468}} \times \left( {1.247{\mkern 1mu} \,{\rm{in}}\,{\mkern 1mu} {\rm{male}}} \right) \times \left( {1.07\,{\mkern 1mu} {\rm{in}}\,{\mkern 1mu} {\rm{PF-}}06439535} \right),$$

where BWT is the baseline body weight.

The parameter estimates for CL and *V*_1_ were 0.0113 L/h and 2.99 L, respectively, for a typical 71-kg female patient with NSCLC receiving bevacizumab-EU. The CL and *V*_1_ were 26.2% and 24.7% higher, respectively, in males than in females; thus, the estimated CL and *V*_1_ were 0.0143 L/h and 3.73 L, respectively, for a typical 71-kg male NSCLC patient receiving bevacizumab-EU. As shown in Fig. 2b, the final model adequately accounted for the effect of sex on CL and *V*_1_, as no significant trend was observed.

The final model was bootstrapped using a resampling approach to evaluate stability of the final model and estimate CIs for all parameters. The non-parametric bootstrapped median and 95% CI values were consistent with the final parameter estimates (Table 2). The 95% CIs for the effect of drug product on both CL and *V*_1_ included unity, providing support that there were no appreciable differences in CL or *V*_1_ between the PF-06439535 and bevacizumab-EU drug products in patients with NSCLC.

**Fig. 4:**
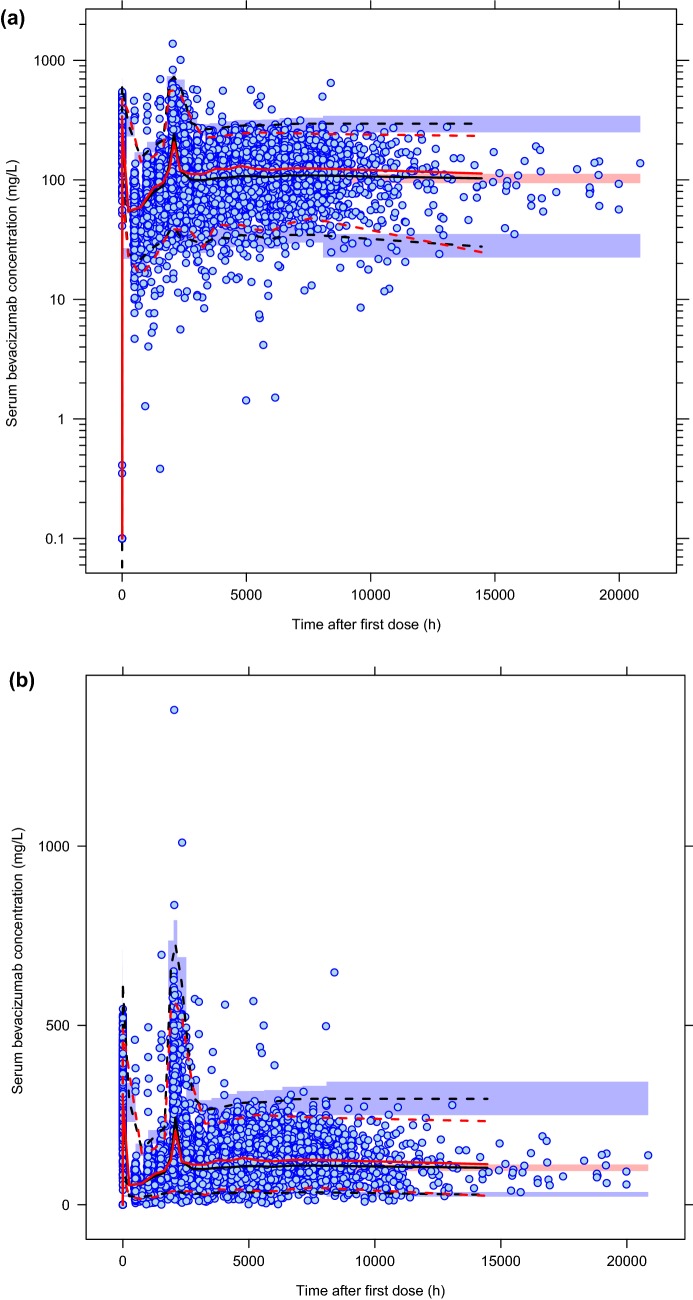
Visual predictive check for the final PF-06439535 and bevacizumab-EU model. The *Y*-axis of plot **a** is presented on the log scale and that of plot **b** is presented on the linear scale. Blue circles represent the observed data and the red lines represent the median (solid line), 2.5th percentile (lower dashed line), and 97.5th percentile (upper dashed line) of the observed data. For the 1000 simulated trials, the median, 2.5th percentile, and 97.5th percentile of simulated concentrations were calculated for each time bin and are presented by black lines. The 95% CIs for the simulated median and each percentile are shown by light pink and light blue shaded areas, respectively. *Bevacizumab*-*EU* reference bevacizumab sourced from the European Union, *CI* confidence interval

Lastly, the predictive performance of the final model was evaluated using a VPC approach for model validation. In general, the model simulation successfully reproduced the observed longitudinal bevacizumab concentration–time profiles for the entire patient pool (Fig. 4).

## Discussion

Although population PK analyses have been previously reported for biosimilar products in patients [13] and healthy subjects [14, 15], this is, to the best of our knowledge, the first published population PK analysis for a bevacizumab biosimilar product based on a comparative clinical study in patients with cancer. The current modeling effort was conducted by developing a model for PF-06439535 and bevacizumab-EU utilizing the combined sparse PK data set from a comparative clinical efficacy and safety study, with covariate analysis incorporating a direct quantitative assessment of the impact of drug product (i.e., the biosimilar or the reference product) on PK parameters.

The availability of only sparse PK data was a limitation of the current analysis, and it presented some challenges. For example, IIV was unable to be incorporated on *V*_2_ or *Q* because of the large relative standard error for these peripheral compartment PK parameters, which was likely a result of the paucity of the data. Nevertheless, the model-based analysis accommodated the use of sparse sampling for the estimation of the central compartment PK parameters, CL and *V*_1_, with the expectation that the impact of sparse sampling on PK would be similar for the reference product and biosimilar.

Consistent with previous reports for reference bevacizumab [1, 11, 12], a two-compartment PK model with first-order elimination from the central compartment adequately described the PK data for PF-06439535 and bevacizumab-EU. The PK parameters were estimated with a sufficient level of certainty and identifiability. The CL and *V*_1_ for a typical 71-kg female patient with NSCLC receiving bevacizumab-EU were 0.0113 L/h and 2.99 L, respectively. These values are comparable to CL and *V*_1_ estimates previously reported for reference bevacizumab [1, 2, 11, 12]. For example, Lu et al. [12] estimated CL and *V*_1_ for a typical female patient with solid tumors as 0.00863 L/h and 2.39 L, respectively. An analysis by Han et al. [11] yielded population estimates for CL and *V*_1_ for a typical 70-kg patient with cancer of 0.0086 L/h and 2.68 L, respectively. Similarly, the US prescribing information for reference bevacizumab [2] reports mean CL and *V*_1_ values of 0.00958 L/h and 2.9 L, respectively. In the current work, the IIV (expressed as a coefficient of variation) for CL and *V*_1_ in the final model was moderate, at 29.5% and 34.2%, respectively. The diagnostic plots and VPC demonstrated adequate fit and predictive performance of the final model.

This analysis identified baseline body weight and sex as significant covariates influencing both CL and *V*_1_, similar to previous findings for reference bevacizumab [1, 2, 11, 12]. The effects of baseline body weight on CL and *V*_1_ were well described in the structural model using a power function, with the exponents estimated as 0.354 and 0.468, respectively. These exponent estimates are similar to those previously published by Lu et al. [12] (0.368 and 0.411, respectively) and Han et al. [11] (0.589 and 0.470, respectively) for reference bevacizumab. After correcting for baseline body weight, our CL and *V*_1_ estimates were approximately 26% and 25% higher, respectively, in males, which is again similar to literature values for reference bevacizumab [1, 2, 11, 12].

Other factors previously identified as influencing the PK of reference bevacizumab, such as tumor burden (measured as the longest tumor diameter in the current study) [1, 2], baseline alkaline phosphatase [11, 12], and baseline albumin [1, 11, 12], were not identified as significant covariates in our analysis. One possible explanation is that the impact of these covariates on PK was relatively low compared with the impact of baseline body weight and sex, and thus may not have been identifiable in this analysis using sparse data from a single study.

The previous PK similarity study (Study B7391001) in healthy subjects [7] demonstrated PK similarity between PF-06439535 and bevacizumab reference products sourced from the EU and US. In the current analysis, drug product was not identified as a significant covariate on CL or *V*_1_. In addition, the 95% CIs of the effect of drug product on CL and *V*_1_ encompassed unity, providing further support for a lack of impact of drug product on these PK parameters.

Our results support the use of a model-based analysis as a supplement to the standard statistical bioequivalence approach used for PK similarity evaluations in the development of biosimilars. The use of a non-linear mixed-effects population modeling approach facilitated the inclusion of sparse data to detect potential differences in PK between PF-06439535 and reference bevacizumab. Nevertheless, the application of this approach can be limited by factors such as data robustness and complexity of the model structure. The success of the current analysis can be partially attributed to the linear PK of bevacizumab and the extensive steady-state PK data collection in the study. In a population PK/PD analysis of recombinant human epoetin alfa and the biosimilar HX575 [14], for example, it was noted that, because of the complexity of the PK/PD model, control of random effects was not straightforward, and this presented challenges in statistical comparison for similarity assessment. Overall, model-based similarity results need to be interpreted with caution and on a case-by-case basis.

The current exploratory population PK analysis, even though not designed for the purpose of demonstrating PK similarity, did not reveal any appreciable differences between PF-06439535 and bevacizumab-EU in terms of CL and *V*_1_. In conclusion, the results of our population PK analysis, conducted using sparse data from patients with NSCLC, provide additional support for the demonstration of PK similarity between PF-06439535 and reference bevacizumab in healthy subjects in Study B7391001.

## Electronic supplementary material

Below is the link to the electronic supplementary material.

Supplementary material 1 (PDF 228 kb)

## Data Availability

Upon request, and subject to certain criteria, conditions and exceptions (see https://www.pfizer.com/science/clinical-trials/trial-data-and-results for more information), Pfizer will provide access to individual de-identified participant data from Pfizer-sponsored global interventional clinical studies conducted for medicines, vaccines, and medical devices (1) for indications that have been approved in the US and/or EU or (2) in programs that have been terminated (i.e., development for all indications has been discontinued). Pfizer will also consider requests for the protocol, data dictionary, and statistical analysis plan. Data from Pfizer trials may be requested 24 months after study completion. The de-identified participant data will be made available to researchers whose proposals meet the research criteria and other conditions, and for which an exception does not apply, via a secure portal. To gain access, data requestors must enter into a data access agreement with Pfizer.
