# Supplementary material for: Population pharmacokinetic modeling of PF-06439535 (a bevacizumab biosimilar) and reference bevacizumab (Avastin®) in patients with advanced non-squamous non-small cell lung cancer
Source: Cancer Chemother Pharmacol. 2019 Nov 26;85(3):487–99. doi: 10.1007/s00280-019-03946-8 (PMC7036079; doi:10.1007/s00280-019-03946-8)
Supplement: Supplementary file 1 — Supplementary material 1 (PDF 228 kb) [file 280_2019_3946_MOESM1_ESM.pdf]

Online Resource

Article title: Population pharmacokinetic modeling of PF-06439535 (a bevacizumab biosimilar) and reference bevacizumab (Avastin®) in patients with advanced non-squamous non-small cell lung cancer

Journal: Cancer Chemotherapy and Pharmacology

Authors and affiliations: Cheryl SW Li[1], Kevin Sweeney[2]*, Carol Cronenberger[3]

[1] Clinical Pharmacology/Pharmacometrics, Pfizer Global Product Development, Pfizer Inc., 300 Technology Square, Cambridge, MA 02140, USA

[2] Clinical Pharmacology/Pharmacometrics, Pfizer Global Product Development, Pfizer Inc., Eastern Point Road, Groton, CT 06340, USA

[3] Clinical Pharmacology/Pharmacometrics, Pfizer Global Product Development, Pfizer Inc., 500 Arcola Road, Collegeville, PA 19426, USA

***Correspondence:** Kevin Sweeney. Email: kevin.sweeney@pfizer.com. Tel: +1-860-441-6041.

Table S1 Parameter estimates from the PF-06439535 and bevacizumab-EU base model

Parameter	NONMEM results OFV = −8096.083		
	**Estimate**	**SE**	**Shrinkage (%)**
V_1 (L)	2.57	0.190	--
V_2 (L)	7.14	0.314	--
CL (L/h)	0.0134	0.000196	--
Q (L/h)	0.791	0.0783	--
BWT effect on V_1	0.796	0.00914	--
BWT effect on CL	0.476	0.0333	--
ω^2_{V1}	0.316	0.0824	29.5
ω^2_{CL}	0.0995	0.00738	7.44
Residual additive error	0.332	0.0138	5.70

Parameters that were not applicable are represented with double dashes.

Bevacizumab-EU=reference bevacizumab sourced from the European Union; *BWT*=body weight; *CL*=systemic clearance; *NONMEM*=nonlinear mixed-effects modeling; *OFV*=objective function value; *Q*=intercompartmental clearance; *SE*=standard error; V_1=volume of distribution in the central compartment; V_2=volume of distribution in the peripheral compartment

Table S2 Summary of covariate evaluation steps from stepwise covariate modeling

**Steps**	**Round #**	**PF-06439535 and bevacizumab-EU**			
		**Covariate**	**Base OFV**	**ΔOFV (drop)**	***P* value**
Base model	N/A	None	−8096.08282	N/A	N/A
Forward selection	1	Sex – CL	−8096.08282	77.38217	1.41e-18*
	1	Sex – V_1	−8096.08282	25.56913	4.27e-07*
	1	Drug product – CL	−8096.08282	0.73403	0.391580
	1	Drug product – V_1	−8096.08282	2.55325	0.110070
	2	Sex – V_1	−8173.46499	28.14694	1.12e-07*
Full model	N/A	Sex – CL<br>Sex – V_1			
Backward elimination	1	Sex – CL	−8201.61192	−79.95996	3.82e-19*
		Sex – V_1	−8201.61192	−28.14694	1.12e-07*
Final model	N/A	Sex – CL<br>Sex – V_1			

*P<0.05 in the forward selection step or P<0.001 in the backward elimination step.

Bevacizumab-EU=reference bevacizumab sourced from the European Union; *CL*=systemic clearance; *N/A*=not applicable; *OFV*=objective function value; V_1=volume of distribution in the central compartment
